# Recent advances in hydrogels-based osteosarcoma therapy

**DOI:** 10.3389/fbioe.2022.1042625

**Published:** 2022-10-12

**Authors:** Hao Tian, Ronghui Wu, Na Feng, Jinrui Zhang, Jianlin Zuo

**Affiliations:** ^1^ Department of Orthopedics, China-Japan Union Hospital of Jilin University, Changchun, China; ^2^ Department of Dermatology, China-Japan Union Hospital of Jilin University, Changchun, China; ^3^ Department of Laboratory Medicine, Nanfang Hospital, Southern Medical University, Guangzhou, China; ^4^ State Key Laboratory of Magnetic Resonance and Atomic and Molecular Physics, National Center for Magnetic Resonance in Wuhan, Innovation Academy for Precision Measurement Science and Technology, Chinese Academy of Science, Wuhan, China

**Keywords:** osteosarcoma, bone repair, hydrogels, OS suppression, bone regeneration

## Abstract

Osteosarcoma (OS), as a typical kind of bone tumors, has a high incidence among adolescents. Traditional tumor eradication avenues for OS such as chemotherapy, surgical therapy and radiation therapy usually have their own drawbacks including recurrence and metastasis. In addition, another serious issue in the treatment of OS is bone repair because the bone after tumor invasion usually has difficulty in repairing itself. Hydrogels, as a synthetic or natural platform with a porous three-dimensional structure, can be applied as desirable platforms for OS treatment. They can not only be used as carriers for tumor therapeutic drugs but mimic the extracellular matrix for the growth and differentiation of mesenchymal stem cells (MSCs), thus providing tumor treatment and enhancing bone regeneration at the same time. This review focuses the application of hydrogels in OS suppression and bone regeneration, and give some suggests on future development.

## Introduction

Osteosarcoma (OS), as a well-known primary bone tumor, involves the invasion of tumors into bone tissue and often occurs in children and adolescents (Mann et al., 2022). Reports suggested that OS has become the second leading cause of death among young cancer patients, especially to the stage of tumor lung metastasis (Siclari and Qin, 2010; Roessner et al., 2021). OS patients may suffer from disability and even death, eliciting heavy blows and losses to the society. Although developing quickly, there are often no obvious clinical signs or severe pain in the early stage of OS. Therefore, it’s critical but tricky for the diagnosis and treatment of OS. To date, clinical treatment strategies include allogeneic bone transplantation and mechanically processed prosthesis (Gianferante et al., 2017; Simpson and Brown, 2018). However, defects such as insufficient allogeneic bone sources and poor biocompatibility severely limit their applications. Besides, most OS can be clean up by surgical intervention, but usually fails to completely ablate the tumor, thus causing post-surgery recurrence and metastasis (Lin et al., 2015; Chen et al., 2017; Friesenbichler et al., 2017; Haghiralsadat et al., 2017; Liu et al., 2021; Wang et al., 2021; Xu et al., 2022). Thus, to avoid postoperative recurrence and metastasis as much as possible, chemotherapy and radiotherapy are combined after surgery. Unfortunately, radiotherapy is reluctant to exert effect in OS and OS is susceptible to chemotherapy resistance (Bohnke et al., 2007; Campbell, 2009; Gianferante et al., 2017; Shoag et al., 2019; Pattee et al., 2020). What’s more, the patients receiving chemotherapy often suffer from side effects including hair loss and vomiting, which will obviously decrease the quality of life (Bosma et al., 2018). At the same time, patients suffered from OS and surgical resection will have bone defects, eliciting acute pain and disability. Thus, implementing the development of OS therapy is a pretty tough work. Correspondingly, innovative and effective methods are urgently needed to guide the therapy of OS in clinical problems (Liao et al., 2021a).

As discussed above, it’s vital to ensure the complete resection of OS after surgery but remains difficulty. Besides, radical resection is dangerous because of the complex anatomical structure and blood vessels in the bone tissues (Wang et al., 2018a; Liu et al., 2019; Wang et al., 2019; Zhang et al., 2019; Yang et al., 2020a; Pan et al., 2020). Along with the development of biotechnology and nanotechnology, novel alternative strategies with less side effects are developed. Specifically, photothermal therapy (PTT) is becoming a promising method that can covert near-infrared (NIR) light into thermal damage in tumor tissues (Chu and Dupuy, 2014; Chen et al., 2016; Liu et al., 2019; Wang et al., 2020; Xu et al., 2021), rejecting tumor region without damaging other organs or tissues (Xing et al., 2016; Shan et al., 2018; Pan et al., 2019; Hou et al., 2020; Jiang et al., 2020). PTT is based on various nanoparticles such as gold (Li Volsi et al., 2017; Mahmoodzadeh et al., 2018; Liao et al., 2019), carbon (Du et al., 2019; Farzin et al., 2019; Guo et al., 2022; Zhang et al., 2022) and copper nanomaterials (Liu et al., 2018). For example, PTT using gold nanoparticles has desirable therapeutic efficacy for prostate cancer in clinical trials (Rastinehad et al., 2019; Taneja, 2020). However, these nanomaterials usually have unsatisfactory biocompatibility and limited bioavailability. Thus, an appropriate carrier is needed to avoid these defects of nanoparticles and benefit their biological applications. More importantly, bone metabolism is becoming unbalanced because of the invasion of tumors into bone, leading to bone defect that is difficult to repair itself (Velasco et al., 2015; Wang et al., 2018b; Khajuria et al., 2018; Zhang et al., 2018). Therefore, therapeutic drugs, growth factors and/or stem cells are urgently needed. Benefiting from the continuous development in biomaterials science, bone tissue engineering scaffolds become a fascinating material with great hope to bone regeneration (Gong et al., 2009; Ni et al., 2014; Li et al., 2018a; Shi et al., 2019). Among various tissue engineering scaffolds, hydrogels with excellent bioactivity, biocompatibility and biodegradability have attracted much attention of researchers. Hydrogels are three-dimensional porous mesh gel with abundant water absorbance (Xu et al., 2020). It can not only afford a vehicle for tumor therapeutic drug but mimic the extracellular matrix (ECM) for the growth and differentiation of mesenchymal stem cells (MSCs), thus providing tumor treatment and enhancing bone regeneration at the same time (Chen et al., 2020). Moreover, when integrating hydrogels with other drug delivery systems such as liposomes and microspheres, carriers with better performance can be created by synergism.

**SCHEME 1:**
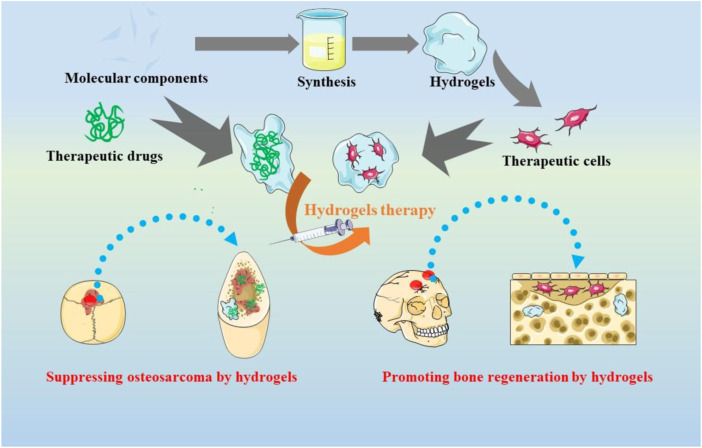
The applications of hydrogels-based therapeutics for suppressing the tumors and promoting bone regeneration in osteosarcoma treatment.

Herein, we discussed the recent advances in the use of hydrogels to achieve OS therapy, with emphasis on suppressing the tumors and Promoting bone regeneration (Scheme 1). We believe that This review will provide a useful reference for hydrogels-based OS therapy and the field of complex diseases to combine tumor therapy and tissue engineering.

## Suppressing the tumors by hydrogels

The conventional therapy strategies for OS comprise the combination of chemotherapy with surgical methods (Dou et al., 2013). Chemotherapy for OS began in the 1970s, which includes doxorubicin (DOX), cisplatin et al. Despite great expectations are expected in chemotherapy, the overall efficacy of which are no more than 60% (Lai et al., 2007) attributing to the high toxic effects of chemotherapy and drug resistance in secondary cancer. Therefore, it’s crucial to construct an artificial implant for the local administration and controlled-release of chemical drugs (Wu et al., 2018).

Several researches have demonstrated that hydrogels are capable of treating tumors due to their porous structure and versatile biocompatibility. It’s acceptable to administer therapeutic drugs or functional cells into the resected OS area with the help of hydrogels (Yang et al., 2020b). With the advantages of providing continuous drug release for tumor illumination, hydrogels encapsulated with drugs can afford localized tumor therapy, replacing systemic chemotherapy administered intravenously (Zheng et al., 2017; Yang et al., 2018; Chen et al., 2019; Peng et al., 2022). As an example, chitosan-based hydrogels are designed for therapeutic agents and cell delivery for tumor therapy (Pan et al., 2019). Besides, thermoresponsive hydrogel based on PEG-g-chitosan (PCgel) can benefit T lymphocyte infiltration into the gel and allow a sustainable release of cells (Jiang et al., 2020). Further, reports have suggested the gelatin gel for the release of anti-carcinogenic drugs (Hu et al., 2018). Wu et al. united gelatin methacryloyl (GelMA) hydrogel with gemcitabine hydrochloride loaded liposomes for OS ablation, which exhibited desirable properties in antitumor and sustained release (Wu et al., 2018). Specifically, the hydrogel system showed feasible application in eradicating OS *in vivo* by MG63-bearing mice.

**FIGURE 1:**
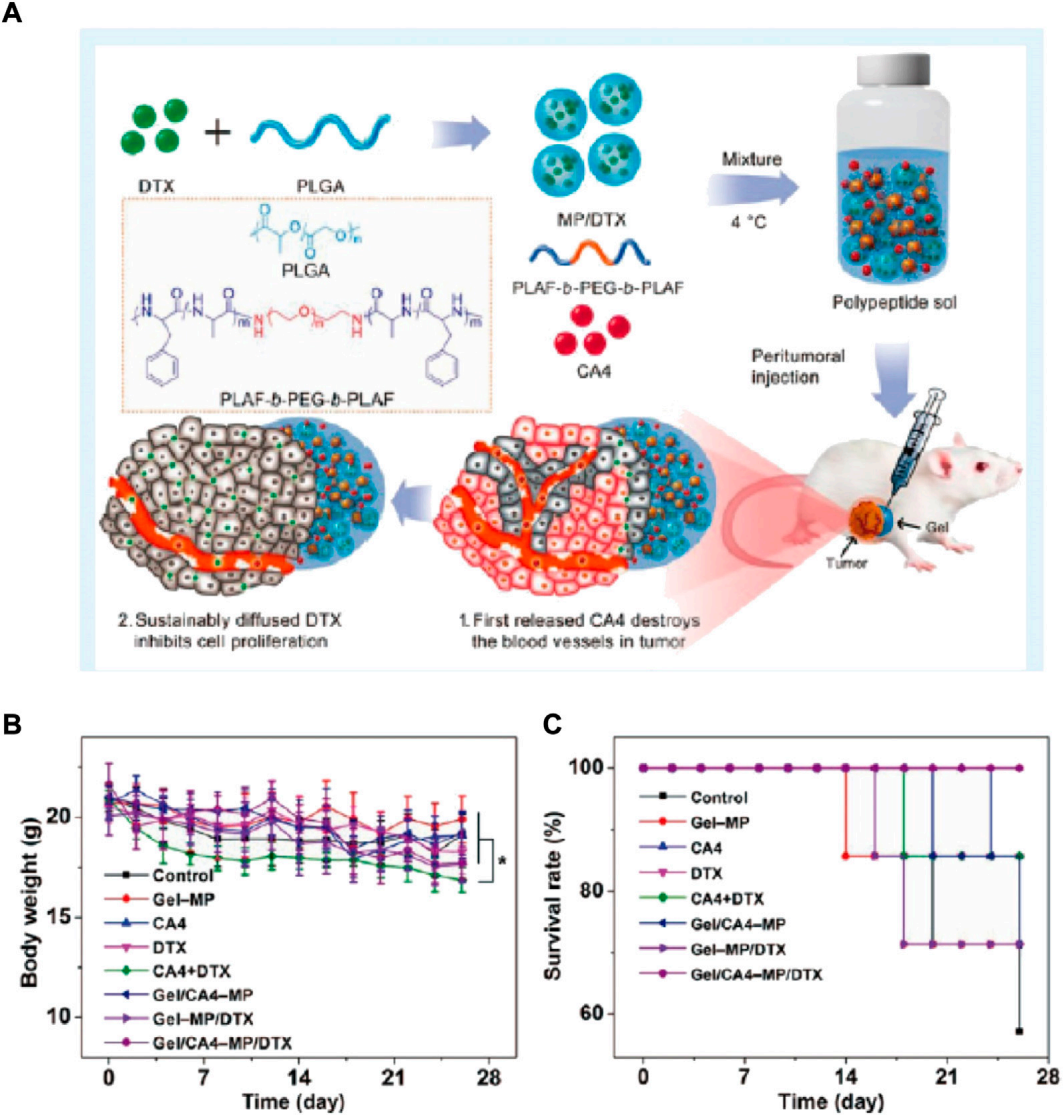
**(A)** Sequential Drug Delivery with Gel-MP Construct, Showing Preparation of Gel/CA4−MP/DTX for Two-Pronged Locally Synergistic Chemotherapy of Osteosarcoma. **(B)** Variations of body weight and survival rate. **(A)** Change of body weight and **(B)** survival rate of K7 osteosarcoma-grafted mice after treatment of PBS as control, Gel−MP, CA4, DTX, CA4+DTX, Gel/CA4−MP, Gel−MP/DTX, or Gel/CA4−MP/DTX. [Data were represented as mean ± SD (*n* = 10; (*) *p* < 0.05)].

Because of the complexity and diversity of tumor pathogenesis, the effect of single chemical drug may be compromised. Thus, synergistic chemotherapy is needed to solve the problem. For instance, Combretastatin A-4 (CA4) are able to bind the tubulin of endothelial cells, disturbing the formation of blood vessel and ultimately, eliciting tumor necrosis through inhibiting the supply of oxygen and nutrients (Perez-Perez et al., 2016). Unfortunately, CA4 can only work on the internal tumors with rich vascular, but often fail to treat the edge of the tumor tissues. Nevertheless, the peripheral tumor tissues are sensitive to traditional drugs like DOX and docetaxel (DTX). For this issue, Zheng et al. developed an injectable thermosensitive hydrogel system for the co-encapsulation and sequential release of CA4 and DTX (Figure 1A) (Zheng et al., 2017). CA4 was released preferentially, which could damage the neovascularization system and inhibit the exchange of nutrients. The followed release of DTX could clean up the surface cells of tumor tissues and lead to apoptosis of the tumor (Figures 1B,C). Likewise, Sun et al. co-loaded Oxaliplatin (OXA) and Alendronate (ALN) onto mPEG45-PLV19 thermosensitive hydrogel (Sun et al., 2020). OXA is a widely accepted anticancer drug, which can induce immunogenic death (ICD) for tumor elimination. ALN have bone affinity as well as the effect of inhibiting bone destruction. Studies found that the system could inhibit the progress of OS and prevent tumor lung metastasis.

**FIGURE 2:**
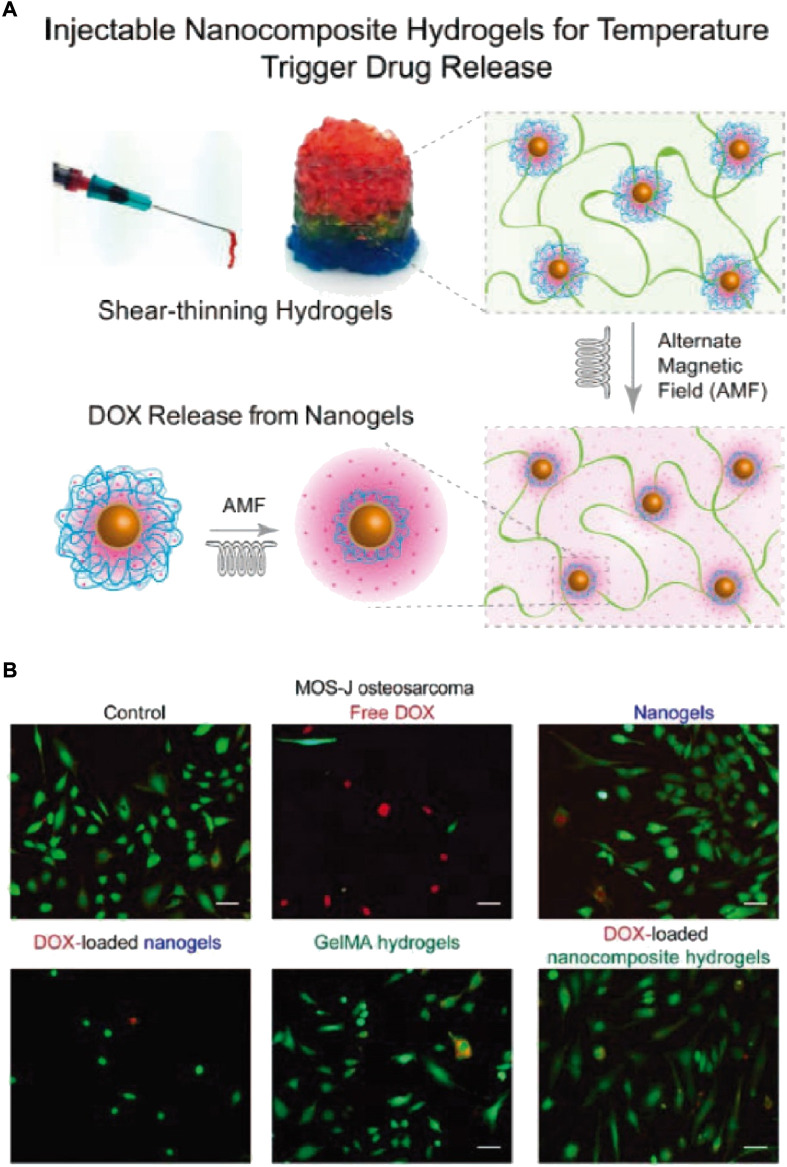
**(A)** Schematic depiction of nanocomposite hydrogels fabrication that can be injected for drug release in response to stimuli. **(B)** MOS-Js, live or dead staining indicated more dead cells upon exposure with free DOX than nanogels and nanocomposites loaded DOX (Scale bar 100 μm).

Currently, the OS treatment regimen is mostly DOX, methotrexate (MTX), and cisplatin (CDDP) based. The clinical efficacy of this regimen was proved by the systemic administration of the aforementioned drugs (Bielack et al., 2009). Ma et al. successfully established a system based on poly (L-lactide-*co*-glycolide)-poly (ethylene glycol)-poly (lactide-*co*-glycolide) (PLGA-PEG-PLGA) for delivering CDDP, MTX, and DOX (Ma et al., 2015). In the human osteosarcoma model of nude mice, the triple-delivery system could induce enhanced tumor apoptosis, displaying high tumor suppression efficacy. Furthermore, the evaluation of alteration in mice’s bodies and their organs histological analysis in *ex vivo* experiment revealed less toxic effects and obvious organ damage after localized treatment. Therefore, local co-delivery of CDDP, MTX, and DOX *via* thermos-sensitive hydrogels might be a promising option for better osteosarcoma treatment. “Smart” hydrogels are novel biomaterials that are influenced by external stimuli. Thus, multiple investigations have been carried out to determine the scope of bio-medical implementations, for instance, regenerative engineering and therapeutic delivery. Jalili et al. established nano-engineered hydrogel comprising poly (NIPAM-co-AM)/MNPs, for local and on-demand injection for delivering drugs (doxorubicin (DOX)) (Figure 2A). In this investigation, shear-thinning hydrogels capable of self-recovering were engineered by manipulating gelatin methacrylate (GelMA) network’s crosslinking density. Prior to this crosslinking GelMA pre-polymer solution was mixed with DOX-loaded Poly (NIPAM-*co*-AM)/MNP nanogels (GelMA/(poly (NIPAM-*co*-AM)/MNPs)). The magnetic field and temperature-dependent DOX release from (GelMA/(poly (NIPAM-*co*-AM)/MNPs)) were evaluated. Lastly, the efficacy of this new form of DOX-carrying drug on pre-osteoblast and osteosarcoma cells was investigated *in vivo* (Figure 2B).

**FIGURE 3:**
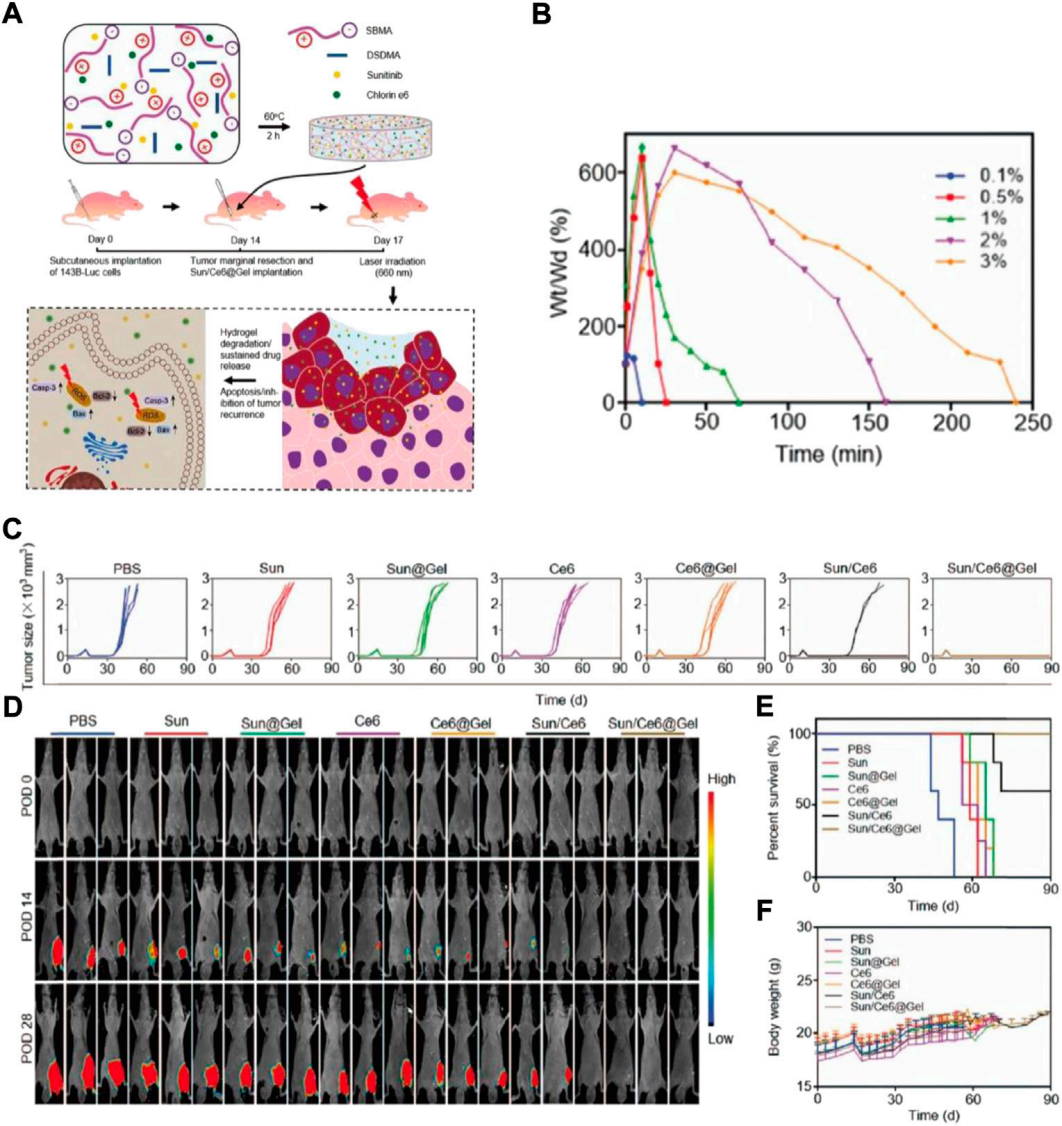
**(A)** Schematic depiction of Sun/Ce6@Gel synthesis and Sun and Ce6 synergistic antitumor impact in inhibiting mice tumor recurrence. Sun/Ce6@gel was generated by fusing Ce6, Sun, DSDMA, and SBMA for 2 h, and was administered in the residual cavity, thereby it inhibited tumor relapse post osteosarcoma marginal eradication. Casp-3: caspase-3. The degeneration and DSDMA–SBMA hydrogels release data. **(B)** Hydrated DSDMA–SBMA hydrogels weight alternations in the solution of PBS supplemented with DTT (100 mM). **(C)** Individual tumor growth kinetics during the treatment. **(D)** *Invivo* nude mice tumor recurrence bioluminescence images. POD, postoperative days. **(E)** Survival rates **(F)** Mice’s body weights were measured, and the effective doses of Sun and Ce65 were selected as 5mg and 1 mg per kg, respectively.

Currently, the therapeutic drug’s maximum tolerated dose (MTD) is not only important for determining the formulation’s concentration, but also for predicting its success in the clinical setting (Kim et al., 2010; Ranade et al., 2014). Even though the MTD of some drugs showed improvement to some extent through the polymer-regulated delivery mechanism, the MTD of drugs that are delivered by hydrogel still needs additional research. For localized OS treatment, Yang et al. used thermo-sensitive hydrogel to incorporate DOX into the poly (D,Llactide-co-glycolide)-poly (ethylene glycol)-poly (D,L-lactide-co-glycolide) (PLGA-PEG-PLGA) (Yang et al., 2018). The PLGA-PEG-PLGA triblock copolymer was successfully prepared and proved by 1H NMR. Furthermore, hydrogel characters, including rheological evaluation, sol-gel phase transition, and drug release in the *in vitro* experiment were studied. The DOX-packed hydrogel’s cytotoxicity was evaluated *in vitro*, in K-7 (mouse osteosarcoma cancer) and Saos-2 (human osteosarcoma cancer) cells. Lastly, the DOX-loaded hydrogel’s antitumor efficacy was determined *in vivo* in the K-7 mice tumor model. DOX-loaded hydrogel’s systemic toxicity and the safety of its local delivery were assessed by mice’s organ pathological analysis and their survival rate. Similarly, Yu et al. suggested a procedure for local Sun and chlorin e6 (Ce6) delivery by zwitterionic redox-responsive hydrogels for preventing the relapse of osteosarcoma (Figure 3A) (Yu et al., 2020). Yu synthesized hydrogels using a redox-responsive cross-linker (DSDMA), in which drugs Ce6 and Sun were introduced, a complex called Sun/Ce6@Gel (Figure 3B). This composite was administered in the residual cavity instantly post tumor eradication. Ce6 and Sun are liberated from the zwitterionic hydrogels because of redox sensitivity post-implantation at the surgery site (Figure 3C). This should result in reduced anti-apoptotic and increased pro-apoptotic gene expression. The potency of Ce6-and Sun-packed hydrogel as a postoperative osteosarcoma therapy was determined in both *in vitro* and *in vivo* conditions (Figures 3D–F).

## Promoting bone regeneration by hydrogels

**FIGURE 4:**
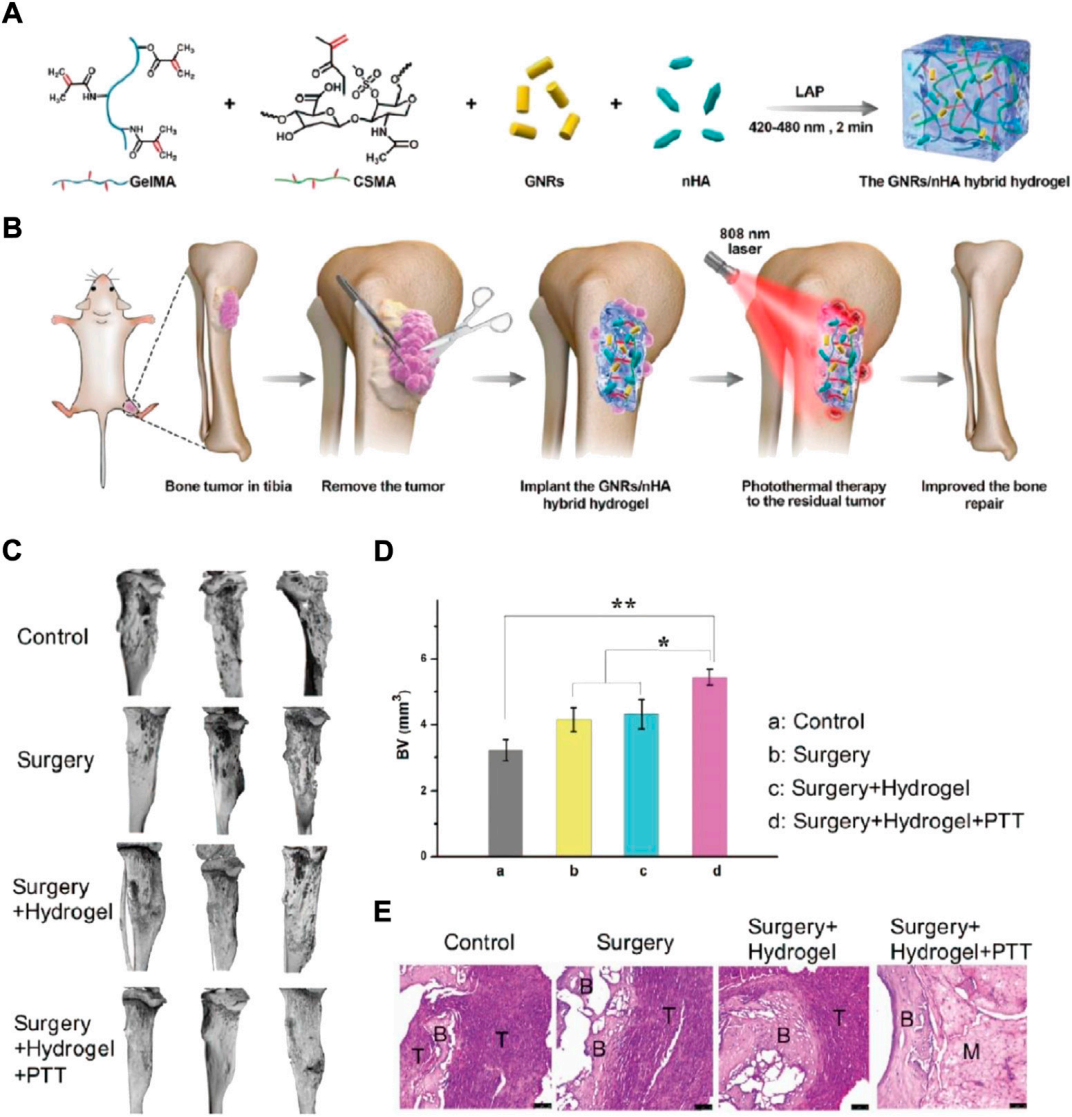
**(A)** The diagrammatic GNRs/nHA hybrid hydrogel preparation. **(B)** The hybrid GNRs/nHA hydrogel application for photothermal treatment and regeneration of bone tumor. **(C)** The micro-CT reconstruction in each group of mice after treatment for 2 weeks; **(D)** The bone volume (BV) parameter in each mice group (*p < 0.05, **p < 0.01); **(E)** The tibia bone tumor H&E stained images of different groups.

Osteosarcoma originates at the epiphyseal end with a rich blood supply. The osteosarcoma effect on bone is huge, and the body takes time to repair or heal itself. Therefore, stem cells, small-molecule, external scaffolds, or growth factor drugs are required (Velasco et al., 2015; Wang et al., 2018b). Recently, computer-assisted digital technology, material mechanics, and bone tissue engineering scaffolds (e.g., 3D printed scaffolds, microspheres, and hydrogels) have progressed a lot with the continuous advancement in the field of biomaterials (Li et al., 2017; Xu et al., 2017). Hydrogels can imitate extracellular matrix (ECM) and improve bone repair by proliferating and differentiating MSCs (Liu et al., 2022). Thus, bone regeneration has widely been studied because of its outstanding osteoinductivity and bio-compatibility, -activity, and—degradability (Feng et al., 2019). Yap et al. established a novel thermoreversible hydrogel scaffold comprising glyoxal (Gx), PLuronic F127, and carboxymethyl hexanoyl chitosan (CA), injected for encapsulating human osteosarcoma MG-63 cells. These hydrogel encapsulated cells proliferated >400% during a 5-day incubation period. The results suggest that F127/CA/Gx hydrogel can envelop cells for tissue engineering (Yap and Yang, 2020). Prosthodontic-inspired photopolymerization stimulated by blue light is a gentle process for initiating the polymerization of monomers (Chen et al., 2020; Downs et al., 2020; Feng et al., 2022), The blue light initiator in the hydrogel system commences hydrogel cross-linking. Human Bone contains 50%–70% inorganic calcium and phosphorus. Nano hydroxyapatite (Ca_10_(PO_4_)_6_(OH)_2_, nHA) has been proven to provide nutrition for bone defects and also help repair bone (Li et al., 2018b; Tan et al., 2021). However, nHA can also inhibit tumors (Barbanente et al., 2021). The hybrid nHA hydrogel is hypothesized to furnish an ECM mimic post osteosarcoma eradication and stimulate bone defect restoration. Liao et al. used light-induced photopolymerization for developing GNRs/nHA hybrid hydrogel (Figure 4A) (Liao et al., 2021b). To generate a biocompatible hydrogel, methacrylate gelatin (GelMA) and methacrylated chondroitin sulfate (CSMA) were used. The nHA and GNRs dissipated easily in the hydrogel. The developed GelMA/CSMA hydrogel, GNRs/nHA hybrid were used for eradicating the residual tumor after surgery *via* PTT and for healing defects after bone tumor surgical resection (Figure 4B). The GelMA/CSMA hydrogel photothermally treated tumors residues left after surgery and repaired the bone deformities in a tibia osteosarcoma mice model (Figures 4C–E).

Osteocytes are crucial for the bone remodeling process, during which the trapped osteoblasts phenotypically alter to mature as osteocytes. The underlying osteocyte mechanisms are still debatable, and it has few study models. E. J. Lee et al. studied how alterations in the mechanical features of bone matrix that lack minerals can affect the phenotypic transformation of osteoblast to osteocyte in a 3D setting *via* bioprinting-based technology called Combing Extrusion printing on Cellulose scaffolds with Lamination (ExCeL) (Lee et al., 2019). Similarly, Vashisth et al. established a biomimetic 3D hybrid scaffold after studying the natural bone architecture with nano-microscale features, favorable porous interconnected structure, and mechanical strength (Vashisth and Bellare, 2018). The key hybrid scaffold constituents are core-sheath nanofibers and hydrogel, which are organized suitably to generate a microenvironment that resembles bone. The core-sheath nanofibers are specifically coiled tightly into a ring to imitate the osteon and reinforced in a hydrogel matrix.

In comparison with traditional biometal scaffolds (like that of titanium and titanium-based alloys), Young’s polyetheretherketone (PEEK) model resembles more human cortical bone, thereby, alleviating osteoporosis and osteonecrosis risk triggered by stress shielding (Wang et al., 2014; Torstrick et al., 2018). Based on this, Yin et al. fabricated a novel and versatile coating made from GelMA hydrogels and TOB-laden MXene nanosheets on an inert orthopedic PEEK material, to eliminate remaining cancerous cells, prevent infection related to bacteria, and guide the regeneration of bone tissues (Yin et al., 2020).

**FIGURE 5:**
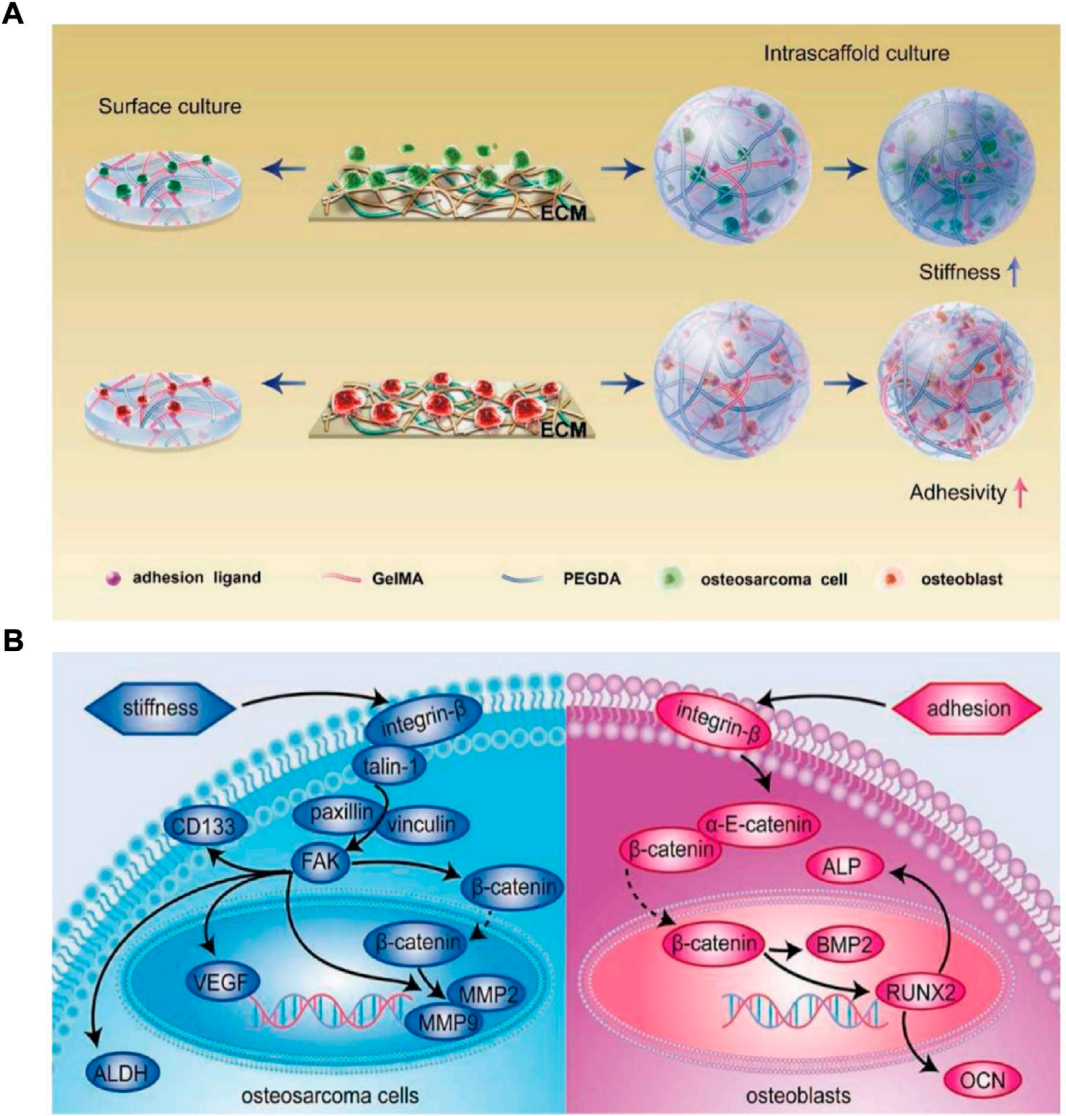
**(A)** Schematic illustration of various anchorage-independent osteosarcoma cells and anchorage-dependent osteoblasts responses to adhesive activity and stiffness of scaffold **(B)** Respond of osteoblasts and osteosarcoma cell’s mechanisms to stiffness and adhesion ligands of ECM, respectively. ECM’s stiffness and not the matrix adhesion influences the growth of osteosarcoma cells by regulating the integrin-induced FA signaling pathway, while osteoblasts are primarily influenced by ECM adhesion ligands *via* the integrin-induced AJ signaling pathway modulation.

How cancerous cells and their normal counterparts have anchorage-dependency and react to the stiffness and adhesion ligand density of the same ECM is still unclear. Jiang et al. analyzed the impact of ECM adhesion ligand density and stiffness on osteosarcoma cells (bone cancerous cells) and osteoblasts (bone-producing cells) *via* poly (ethylene glycol) diacrylate (PEGDA) and GelMA hydrogels (Figure 5A) (Jiang et al., 2019). When osteosarcoma cells were cultured in 3D PEGDA/GelMA hydrogel matrix, they showed high dependence on the stiffness of the matrix by modulating the integrin-induced pathway of focal adhesion (FA), whereas osteoblasts showed high sensitivity toward matrix adhesion ligand density by modulating the integrin-induced pathway of adherens junction (AJ) (Figure 5B). But in the 2D hydrogels surface culture, bone cancerous cells presented a different behavior and showed sensitivity to the matrix adhesion ligand density due to their “forced” attachment to the substrate, similar to anchorage-dependent osteoblasts.

## Conclusion and prospects

Hydrogels are enormous water meshes that characteristically resemble extracellular matrix. These are very porous and have excellent biological compatibility and degradability. They are capable of introducing growth factors that can repair bone defects (Torstrick et al., 2018; Zhang et al., 2018). Therefore, they are good suiters for repairing bone. Literature indicates potential hydrogels application for regenerating bone tissue. For potential bone cancer therapy, these should first be capable of curing tumors. Administering drugs or other molecules directly at the resected tumor site for treatment is highly advised. Hydrogels provide sustainable drug release for tumor eradication (Ali Gumustas et al., 2016; Hu et al., 2020). Some act by delivering the drug directly to the specific system 126. Localized hydrogel therapy for cancer treatment can replace systemic chemotherapy given orally or intravenously (Zheng et al., 2017; Yang et al., 2018; Chen et al., 2019). With the discovery of new hydrogel functions, their implementations are no longer limited to repairing tissues, it has extended to bone repair and tumor eradication (Table 1).

**TABLE 1:** Recent summary of therapeutic strategies for hydrogel osteosarcoma.

Components	Models	Strategies	References
Chitosan	Tumor	Therapeutic drug delivery	33
PEG-g-chitosan	Tumor	Immunotherapy	31
Gelatin methacryloyl	OS	Therapeutic drug delivery synergy therapy	56
Injectable thermosensitive hydrogel	OS	Co-encapsulation and sequential release of CA4 and DTX	61
Poly (NIPAM-co-AM)/MNPs	OS	Local and on-demand injection for delivering drugs [doxorubicin (DOX)]	66
GNRs/nHA	Bone regeneration	Healing defects after bone tumor surgical resection	80
Poly (ethylene glycol) diacrylate (PEGDA) and GelMA	Bone regeneration	Modulating the integrin-induced pathway of adherens junction	86

However, hydrogel’s application in osteosarcoma is limited for the following reasons, first, despite extensive literature research on hydrogels, clinical applications have encountered bottlenecks, and only a few hydrogels have been approved and commercialized (Fan et al., 2022; Li et al., 2022). Additionally, cytotoxicity is often stimulated because of the hydrogel’s inorganic nature and the metal ions involved. Hydrogels are mainly developed from raw materials that are non-essential to organisms. With further investigations solving the aforementioned issues associated with osteosarcoma therapy-related hydrogels, it is expected that a promising candidate might be discovered that would contribute to human health and well-being.
